# Exploring the Intricate Nexus of Sarcopenia and Cognitive Impairment

**DOI:** 10.14336/AD.2023.1013

**Published:** 2023-10-18

**Authors:** Yiwen Xing, Xiaxia Li, Lina Ma

**Affiliations:** Department of Geriatrics, Xuanwu Hospital, Capital Medical University, National Clinical Research Center for Geriatric Disorders, Beijing, China.

**Keywords:** sarcopenia, cognitive impairment, mechanisms, biomarkers, intervention

## Abstract

Sarcopenia, a group of skeletal muscle diseases with high prevalence in older adults, usually manifests as loss of muscle mass and strength, and/or physical performance decline. Cognitive impairment, defined as impaired function in one or more cognitive domains such as memory, language, computation, comprehension, executive, and visuospatial skills, affects the quality of life and social functioning of patients. Both sarcopenia and cognitive impairment are common geriatric syndromes, and the two disorders interact and influence each other. Declining muscle function accelerates cognitive impairment, and cognitive impairment in turn affects muscle strength. Potential common pathological mechanisms between the two include chronic inflammation, mitochondrial dysfunction and oxidative stress, and gut microbiota disorder. Additionally, neuroendocrine connections including testosterone, insulin, and growth factors have important effects on muscle and brain function. Recently, the development of applied metabolomics technologies has shown significant potential in uncovering shared biochemical pathways and exploring potential biomarkers. Exercise, nutritional, and cognitive interventions are significant as nonpharmacologic approaches in the treatment of sarcopenia and cognitive impairment. However, the specific mechanism of interaction between two diseases, biomarkers and effective therapeutic medications still has knowledge gaps that need to be further explored.

## 1. Introduction: Unveiling the Linkages

The global population is increasingly aging. Worldwide, 11% of individuals exceed 60 years of age, and this percentage is expected to reach 22% by 2050 [1]. Aging is characterized by telomere attrition, stem cell exhaustion, deregulated nutrient sensing, epigenetic alterations, dysbiosis, impaired macroautophagy, altered intercellular communication, mitochondrial dysfunction, genomic instability, chronic inflammation, loss of proteostasis, and cellular senescence [2]. As age increases, skeletal muscle aging can manifest as sarcopenia, which is characterized by decreased muscle mass and strength. A study shows that approximately 10-16% of the world's older adult population suffer from sarcopenia [3]. Cognitive impairment (CI) is also a group of age-related diseases that can manifest as mild cognitive impairment (MCI) and dementia. The prevalence of MCI is approximately 6.7-25.2% in older adults [4]. The age-standardized prevalence of dementia is 5-7%, with a higher prevalence in women than in men, and a 244 times higher prevalence of all types of dementia among persons aged 100 years and older than in those aged 50-59 years [5, 6].

Both sarcopenia and CI are age-related conditions, and they influence each other. Current research suggests that sarcopenia increases the risk of CI, and CI also affects muscle function such as gait speed modulation [7, 8]. The prevalence of CI in people with sarcopenia has been reported as 54.4%, which was significantly higher than that in persons without sarcopenia (20.9%) [9]. The prevalence of sarcopenia is significantly higher in patients with dementia (26.4%) than in persons without dementia (8.3%) [10].

Despite the widespread interest in the study of sarcopenia and CI, current research has focused on the interaction between the two diseases, but the common pathophysiological mechanisms, and related biomarkers are unclear. Therefore, this commentary focuses on providing an overview of the common pathological mechanisms of the two diseases, new findings from cross-sectional and longitudinal studies of their interactions, and possible potential biomarkers and interventions.

## 2. Sarcopenia and Cognitive Impairment: Shared Pathophysiological Mechanisms

Sarcopenia and CI are age-related disorders, and they may share some common pathophysiological mechanisms, such as chronic low-grade inflammation, mitochondrial dysfunction and oxidative stress, and gut microbiota disorder.

### 2.1. Chronic inflammation

Chronic inflammation is a condition in which certain psychological, social, and environmental factors prevent acute inflammation from subsiding, leading to a state of sterile, low-level, systemic chronic inflammation [11]. Chronic inflammation is a feature of human aging and can manifest as elevated levels of inflammatory markers, such as interleukin-6 (IL-6) and C-reactive protein which predict changes in neuronal health as well as body composition [11, 12].

IL-6 and tumor necrosis factor α (TNF-α) are both common pro-inflammatory cytokines, while interleukin-10 (IL-10) is an anti-inflammatory cytokine, and an imbalance in the ratio of these inflammatory cytokines *in vivo* can affect muscle function. Sarcopenia is usually accompanied by elevated levels of pro-inflammatory indicators [13]. During muscle atrophy, increased expression of pro-inflammatory cytokines leads to enhanced catabolism in skeletal muscle, while certain proteases (E3 ligase, calpain) and inflammatory pathways (NF-κB) are regulated by pro-inflammatory cytokines and their expression levels are increased to promote protein catabolism, resulting in sarcopenia [14]. Serum IL-10 levels were reduced in patients with sarcopenia, and the sarcopenia severity correlated positively with IL-6 and TNF-α levels [15].

Chronic inflammation plays an important role in CI progression. Compared with a heathy control group, patients with Alzheimer's disease (AD) showed significantly increased peripheral levels of IL-6 and TNF-α [16]. Activation of the IL-6 pathway can also be observed in some regions of the brain in AD mouse models [17]. IL-10 suppresses neuroinflammation and astrocyte/microglia proliferation and enhances cognition and neurogenesis in APP+PS1 mice [18]. Significantly lower levels of IL-10 have also been observed in the sera of AD patients [19].

Thus, both skeletal muscle and cognitive functions are affected by chronic inflammation *in vivo*, and elevated levels of pro-inflammatory cytokines (IL-6 and TNF-α) and decreased levels of anti-inflammatory cytokines (IL-10) may contribute to sarcopenia and CI.

### 2.2. Mitochondrial dysfunction and oxidative stress

Mitochondria are involved in ATP production and metabolic regulation and are important organelles in muscles [20]. Myocyte mitochondrial dysfunction is a major contributor to age-related muscle function degeneration [21]. Patients with sarcopenia can exhibit mitochondrial dysfunction, such as low peroxisome proliferator-activated receptor gamma coactivator 1α/estrogen-related receptor α signaling and downregulation of mitochondrial protease genes [22]. These changes lead to reduced expression of mitochondrial respiratory complexes and decreased nicotinamide adenine dinucleotide levels [22].

Oxidative stress is caused by an imbalance between oxidative and anti-oxidative reactions toward a pro-oxidative state, and is associated with the development of certain diseases [23]. Oxidative stress can be a consequence and/or cause of mitochondrial dysfunction [24]. Oxidative stress influences altered muscle function [25]. Significant increases in oxidative stress markers, such as oxidized glutathione/reduced glutathione ratios in blood and plasma malondialdehyde/4-hydroxy-2,3-nonenal protein adducts have been found in patients with sarcopenia [26].

Oxidative stress has been strongly associated with aging and neurodegenerative diseases [27]. Mitochondrial dysfunction and oxidative stress drive the pathophysiological processes underlying MCI and AD [28]. Mitochondrial DNA mutations, metabolic level disorders, and environmental factors may cause mitochondrial dysfunction, which in turn may result in various pathological changes, such as increased reactive oxygen species denaturation, decreased ATP production, and accumulation of defective organelles [29]. This in turn may trigger or exacerbate oxidative stress, energy deficiency, and ultimately CI and neuronal apoptosis, with the above-mentioned complex series of pathological processes interacting and influencing each other to promote the development of AD [29].

### 2.3. Gut microbiota disorder

Gut microbiota are normal microorganisms that are hosted in the gut for a long period of time and play an important role in maintaining human health. In recent years, the concepts of the muscle-gut and brain-gut axis have been proposed to suggest that gut microbiota are closely related to muscle function, cognitive ability, and metabolic response. The gut microbiota modulates skeletal muscle function and mass in mice[30]. Gut microbes can regulate muscle function through mechanisms such as metabolizing glucose, lipid, proteins, and other substances, influencing levels of inflammation in the body, and neuromuscular connectivity [31]. Disturbances in the gut microbiota can affect muscle condition. Compared with the non-sarcopenia population, patients with sarcopenia had reduced gut microbiota abundance, with *Acetate* and *Agathobacter* positively correlating with gait speed and grip strength, respectively, and *Bifidobacterium* negatively correlating with appendicular skeletal muscle index and grip strength [32]. Supplementation with short-chain fatty acids, prebiotics, and probiotics had a positive impact on improving muscle function and is a potential new treatment for sarcopenia [31].

Gut microbiota modulates cognitive behavior and brain function through the microbiota-gut-brain axis [33]. Bacteria in the gut secrete lipopolysaccharides and amyloid proteins to regulate signaling pathways, and gut microbiota disorder can also alter the permeability of the gut and blood-brain barrier, which have all been implicated in the pathogenesis of AD [34]. Changes in the relative proportions of different microbiota in the gut can affect cognitive function, and supplementation with appropriate amounts of probiotics can improve cognitive performance. A significant increase in *Prevotella* in the gut of middle-aged and older patients with MCI, and improvement in cognitive function scores after supplementation with the probiotic *Lactobacillus rhamnosus* GG was associated with a decrease in the relative abundance of *Dehalobacterium* and *Prevotella* [35].

The microbiota in the gut changes with age. Gut microbiota disorder can alter muscle and cognitive function by affecting substance metabolism, and modulating signaling pathway transduction. Therefore, supplementation with appropriate amounts of probiotics, prebiotics, and other products that modulate the gut microbiota may provide new therapeutic ideas for improving sarcopenia and CI.

## 3. Emerging Findings from Cross-sectional and Longitudinal Studies

### 3.1. Effect of sarcopenia on cognitive function

Declining muscle function affects cognitive ability and increases the risk of CI in older adults. The prevalence rates of MCI in the sarcopenia, probable sarcopenia, and non-sarcopenia groups were 24.2%, 16.5%, and 10.1%, respectively, in older Chinese adults [7]. One cohort from Mexico, with an 8-year follow-up, the prevalence of MCI increased by 0.8% and 1.5% per year in individuals without and with sarcopenia, respectively [36].

A decline in muscle function can accelerate the rate of cognitive decline. Patients with more severe sarcopenia at baseline had a faster rate of cognitive decline during the 5.6-year follow-up in a study from the United States [37]. Grip strength is one of the common measures of muscle strength. Grip strength was associated with MCI in older adults, and this association was particularly strong in older men [38]. In addition, low grip strength is associated not only with overall cognitive function in men but also with execution, attention, and verbal fluency [39], indicating that screening for muscle strength may be useful in predicting CI risk. Developing a proper exercise program to improve muscle strength may help delay the transition to CI in older adults [40].

The series of cross-sectional and longitudinal studies described above suggest that declining muscle function accelerates cognitive decline, and that low muscle strength may be a predictor of the occurrence of CI.

### 3.2. Effect of cognitive impairment on sarcopenia

Decreased cognitive function affects muscle strength and physical activity. The prevalence of sarcopenia is significantly higher in people with dementia compared to those without dementia and is approximately three times higher than in the population without dementia [10]. A prospective study from Brazil found that CI may predict sarcopenia in older adults, and that the presence of CI at baseline, underweight or overweight, and advanced age over 80 years predicted sarcopenia 9 years later [41].

Decreased muscle strength and slower gait speed in the upper and lower extremities can occur in the early stages of AD, whereas patients with moderate AD show decreased muscle strength and mass [42]. Cognitive dysfunction may also affect a patient's gait and balance in the early stages of memory loss [43]. Older adults with MCI have an increased risk of falls as compared to cognitively normal older adults, possibly because of greater variations in stride length and swing time, reduced gait adaptation, and gait control during the transition from fast to slow gait speed [8]. Patients with amnestic MCI (aMCI) may be characterized by a slower and more variable gait speed, and their slower gait speed may be associated with structural changes associated with reduced cortical thickness and gray matter volume of the brain [44]. Therefore, early identification, management, and intervention of CI may help to prevent adverse events, such as falls.

The above studies have revealed a correlation between sarcopenia and CI, with sarcopenia increasing the risk of CI and a decline in cognitive function in turn affecting muscle function. However, the specific mechanisms interaction between sarcopenia and CI have not been fully elucidated. Therefore, it can be further explored by detecting changes in metabolic levels in patients or constructing animal models.

## 4. Neuroendocrine Connections: Hormonal Influences on Muscle and Brain

### 4.1. Testosterone

Testosterone is an androgen that performs important functions in organisms. Testosterone increases protein synthesis by activating metabolic alterations in anabolic muscle pathways (mammalian target of rapamycin, mTOR), which may be associated with an increase in the L-type amino acid transporter 2 channel and neutral amino acid transporter, thereby enhancing the availability of essential amino acids (EAA) [45]. Many older adults have lower than normal testosterone levels. Low levels of free testosterone are associated with a decreased timed up and go test time in men and decreased muscle strength in women at 2 years [46]. Moderate testosterone supplementation in older adult men helps to increase muscle strength and muscle protein synthesis, and supplementation with near-physiological testosterone therapy for 6 months can improve muscle volume and arm and leg strength [47].

Testosterone also has biological significance in the human central nervous system [48], affects neural activity associated with memory and learning, and has a neuroprotective effect during aging [49]. Lower systemic testosterone levels may increase the risk of all-cause dementia or AD [50], possibly because testosterone reduces β-amyloid accumulation [51]. On the other hand, appropriate testosterone supplementation in male patients improves cognition. Testosterone treatment in men with MCI and low testosterone levels modestly improved memory, language function, and depressive symptoms [52]. However, studies on the effects of testosterone supplementation on the cognition of women with CI are limited. One study showed that testosterone treatment significantly improved memory and verbal learning in postmenopausal women [53].

### 4.2. Insulin

The skeletal muscle is an important target organ of insulin. Insulin enhances protein synthesis by regulating the mTOR pathway [54] and has an anabolic effect in muscle, affecting protein metabolism and thus muscle function [55]. Patients with sarcopenia have significantly lower insulin levels [56], and the deficiency of this hormone is associated with a net loss of protein and decreased myoblast activity [54]. Therefore, sarcopenia is also more prevalent among patients with type 2 diabetes mellitus, with a prevalence of 7-29.3% [55].

Insulin also plays an important role in memory and learning in the hippocampal region by regulating synaptic transmission and plasticity [57]. Aging is a factor in the development of insulin resistance [58]. The brains of patients with AD demonstrate insulin resistance and are associated with the pathophysiological processes of AD [59]. Insulin resistance in the brain is accompanied by insulin-like growth factor-1 (IGF-1) resistance and may be associated with insulin receptor substrate-1 dysfunction [60]. Compared with wild-type mice, APP/PS1 mice showed differences in insulin signal transduction and some cognitive fields in their brains [59].

### 4.3. Growth factors

Growth factors are a type of peptides that regulate cell growth. Fibroblast growth factor (FGF) and its receptor regulate muscle and bone mass during the aging process [61]. FGF2 maintains muscle homeostasis, and FGF2 knockout affects age-related alterations in muscle phenotype, affecting forelimb and hindlimb stance and thus potentially altering gait in mice [62]. FGF19 regulates skeletal muscle growth by enlarging muscle fibers, increasing skeletal muscle mass, and preventing skeletal muscle atrophy [63]. Moderate supplementation of FGF19 treatment increases skeletal muscle fibers and reduces sarcopenia complications in chronic kidney disease mice [64]. FGF21 may have a muscle catabolic function; elevated serum levels of FGF21 affect muscle strength and mass, and correlate with the development of sarcopenia [65, 66]. Therefore, the development of FGF-related medicines to improve muscle function has potential therapeutic implications.

Growth factors promote neuronal proliferation and are neuroprotective. FGF-2 is a neurotrophic factor that is expressed in a variety of neuronal cells [67]. A study finds that subcutaneous injection of FGF2 reduces tau pathologies and β-amyloid and improves spatial memory function in APP 23 transgenic mice, with potential applications for the treatment of AD [67]. FGF-23 is mainly synthesized and secreted by osteoblasts and distributed in the hypothalamus and hippocampus in the nervous system [68, 69]. Serum FGF23 levels are positively associated with the risk of developing dementia, suggesting that FGF23-related metabolic pathways may drive the development of dementia [70].

## 5. Metabolomics as a Tool for Insight: Linking Biomarkers

Developments in the application of metabolomics technology offer the possibility of searching for potential biological markers of sarcopenia and CI. Sugars, lipids, and proteins, as important nutrients in living organisms, are metabolized at levels that change under different pathological conditions. Using metabolomics techniques, Kameda et al. identified 22 metabolites as biomarkers for sarcopenia, including metabolites associated with glucose metabolism (pentose phosphate) [71]. Phospholipid levels are equally important for muscle function. Muscle phosphocreatine levels in older adults with sarcopenia are lower than in those without sarcopenia [72]. Disorders in amino acid metabolism are associated with the development of sarcopenia [73]. Serum levels of valeryl-L-carnitine have been independently associated with sarcopenia and may serve as a potential biomarker for this condition [74]. Dihydroxyphenylalanine, α-aminoadipic acid, and citrulline/ornithine levels in males and glutamate levels in females are sex-linked biomarkers of muscle mass loss [75].

Disorders of cholesterol metabolism may affect cognitive function. Mini-Mental State Examination scores combined with hair sterol signatures can be used for predictive diagnosis of CI screening [76]. Bile acids are cholesterol metabolites found in the liver. Bile acids have neuroprotective functions and disorders of bile acid metabolism, and the expression of their different receptors may be biomarkers of the prognosis of cognitive dysfunction-related diseases [77]. In addition, branched-chain amino acids (BCAAs) impair neuronal function, and the levels of BCAAs and their metabolites are increased in patients with AD as compared with those with normal cognitive function [78]. Among them, valine levels were positively correlated with delayed recall scores on auditory verbal learning tests in patients with subjective cognitive decline plus [79].

Factors related to lipid metabolism may improve muscle and cognition function. Daily supplementation with 1-4 g of L-carnitine for 24 weeks improved cognitive function and muscle mass in healthy centenarians [80]. Phospholipids are the main components of cell membranes that maintain cell metabolism and participate in cell signaling [81]. Reduced levels of erythrocyte phospholipids and unsaturated fatty acids may be key factors in the progression of sarcopenia and MCI and may also serve as biomarkers for sarcopenia and/or CI in older adults [82].

## 6. Clinical Implications and Therapeutic Avenues

Both sarcopenia and CI increase the risk of adverse clinical outcomes such as falls, frailty, and decreased physical functioning in older adults, affecting their quality of life. Therefore, early intervention for sarcopenia and CI is necessary to delay or prevent muscle and cognitive decline. In recent years, in addition to pharmacologic treatments, non-pharmacologic interventions have made partial progress in the treatment of sarcopenia and CI.

### 6.1. Exercise interventions

Sedentary or reduced physical activity is common among the older population, with only about one third of persons over 65 years of age engaging in physical activity during their free time [83]. The number and size of muscle fibers decline with age, which affects muscle function. Exercise interventions can improve muscle strength and physical mobility [84-86]. Among them, resistance exercise, which is active movement of muscles against a certain resistance, is an effective way to alleviate sarcopenia. After 16 weeks of elastic band and body weight-based resistance training (three times a week, one hour each time), female patients with sarcopenia over 65 years old had improved grip strength, walking speed, and other indicators [87]. Ten weeks of easy-to-follow resistance exercise in 70-year-olds increased muscle mass and maintained functional strength in patients with pre-sarcopenia [84].

Physical activity is equally positive in improving cognitive function. Mixed physical activity had less impact on overall cognitive functions in patients with MCI, but resistance training had a greater impact on overall cognitive performance in such patients [88]. In addition, physical activity not only improved overall cognitive functioning in patients with dementia but also had a positive impact on non-cognitive symptoms such as falls [88]. Physical activity improved cognitive function by modulating levels of inflammatory cytokines and neurotrophic *in vivo* [89]. In patients with aMCI, 16 weeks of aerobic exercise elevated serum brain derived neurotrophic factor levels and decreased TNF-α, interleukin-15 (IL-15), and insulin levels, furthermore, resistance exercise elevated serum IGF-1 levels and decreased IL-15 levels [89]. Resistance exercise also protected the hippocampal subregion, which is prone to AD degeneration, and this neuroprotection was long-lasting, at least up to 1 year after an exercise intervention, so resistance training had long-lasting benefits in terms of improvements in cognitive functioning levels [90].

### 6.2. Dietary interventions

Rational dietary structure has potential implications for the prevention and treatment of sarcopenia and CI. Among the many dietary patterns, the Mediterranean Diet (MD) is a dietary structure that has received a lot of attention. The MD is characterized by an abundance of vegetables, fruits, olive oil, seafood, moderate intake of dairy products, red wine and poultry, and a low intake of red meat [91]. Adherence to the MD may be protective against sarcopenia and help maintain muscle function and mass. The MD may be beneficial in preventing sarcopenia, and a healthy diet reduced the prevalence of sarcopenia 16 years later [92]. Women with higher MD scores had faster walking speeds, higher lower body muscle quality and short physical performance battery scores, and less loss of total body lean mass and relative skeletal muscle index after 3 years of follow-up [93].

Nutrition may modify neuroinflammatory processes associated with the pathological process of AD, and the MD as an anti-inflammatory dietary pattern may have a neuroprotective function [94]. In one study, an extra-virgin olive oil-rich MD improved cognitive function in participants [95]. In another study, a reduced risk of cognitive decline and dementia was found in participants with higher adherence to the MD pattern in a Mediterranean population [96]. The mechanism may be related to the fact that the MD prevents brain atrophy, and increases total brain volume, total white and gray matter volume, preservation of white matter structures, and structural connectivity in the brain [97, 98].

### 6.3. Cognitive training

Cognitive training is a kind of intervention that applies a variety of cognitive tasks to improve multiple domains of cognitive functioning. Cognitive training combined with a variety of interventions such as exercise and nutrition can help improve muscle function in older adults. In hospitalized older adults, sarcopenia is associated with cognition and physical mobility, so more emphasis should be placed on training for motor-cognitive abilities should be emphasized when developing individualized interventions [99]. Older patients with sarcopenia who were treated with a variety of lifestyle interventions, including cognitive and nutritional interventions, had lower sarcopenia scores at 3- and 6-months post-intervention compared to baseline [100]. In addition, 12 weeks of physical combined with cognitive training improved gait performance, balance, and lower extremity muscle strength in cognitively normal older women [101].

In the early stages of cognitive decline, cognitive interventions can help to improve cognitive performance and delay cognitive decline. Cognitive training in patients with MCI was effective in improving cognitive function and may reduce deterioration in cognitive ability [102]. Cognitive training not only improves a variety of cognitive domains such as language, orientation, and attention in patients with MCI, but also reduces the incidence of unintentional adverse events such as falls and improves the quality of life of older adults [103, 104].

## 7. Gaps in Knowledge and Future Research Directions

There is currently considerable interest in research on sarcopenia and CI. Although a growing number of researchers suggest that there is an interaction between the two diseases, there are still some knowledge gaps that need to be further explored. First of all, most of the studies only focus on the interaction between the two diseases, but the specific mechanism of their interaction has not been fully elucidated. In addition, despite the high prevalence of sarcopenia and CI, early detection is difficult and specific biomarkers are lacking. Sugars, lipids, proteins, and their metabolites are important nutrients, and changes in the levels of metabolites *in vivo* under pathological conditions may reveal common pathological mechanisms associated with sarcopenia and CI. However, metabolite levels are susceptible to conditions such as diet, disease, and nutritional status, leading to differences in biomarkers found in different studies. Finally, both sarcopenia and CI are age-increasing disorders, with symptoms progressively worsening with age and disease progression. However, effective medications and interventions for the treatment of sarcopenia and CI are lacking.

Therefore, high-quality large population cohorts could be further established to longitudinally explore the correlation between the two diseases through long-term standardized follow-up. Specific mechanisms of interaction between the two diseases, as well as potential biomarkers, should be explored by constructing animal models to design experiments on the underlying pathomechanisms. Development of new medicine and safety studies in animal models could be based on identified pathomechanisms and biomarkers, and further randomized controlled trials in populations are necessary to explore effective treatments and interventions.

## 8. Ethical and Practical Considerations

As mentioned previously, well-designed animal and population-based studies are necessary to further explore the pathomechanisms, potential biomarkers, and interventions for sarcopenia and CI. Animal welfare should be taken into account when conducting animal experiments, in line with the 3R ethical principles of replacement, reduction, and refinement for laboratory animals [105]. Designing interventions in populations may face challenges such as participants' rights to informed consent, choice, and privacy, and the use of reasonable placebos. Therefore, similar to interventions in other fields, randomized controlled clinical trials on sarcopenia and CI should emphasize the three basic ethical and moral requirements of informed consent of participants, assessment of the risk-benefit ratio, and equitable distribution of research benefits and burdens [106]. In actual research work, it is necessary not only to scrutinize the ethical information at the initial stage of the research, but also to carry out long-term supervision.

## 9. Conclusion: Embracing Complexity for Holistic Well-being

There is an interaction between sarcopenia and CI. Decreased muscle function affects cognitive ability and increases the risk of CI, while decreased cognitive function also affects muscle strength, gait, and other physical activities. There are some common pathologic mechanisms between the two diseases. Elevated levels of pro-inflammatory cytokines and decreased levels of anti-inflammatory cytokines, mitochondrial dysfunction and oxidative stress, as well as gut microbiota disorder all have an impact on muscle and cognitive function. In addition, neuroendocrine connections including altered levels of testosterone, insulin, and growth factors *in vivo* can also affect muscle and brain function. Developments in the application of metabolomics provide new ideas and methods for finding potential biomarkers for sarcopenia and CI. Exercise, nutritional, and cognitive interventions as non-pharmacological treatments have positive effects in improving muscle and cognitive function. In future studies, multidisciplinary collaborative intervention models are important to address the double burden of sarcopenia and CI.
